# Mitochondrial sequences reveal a clear separation between Angolan and South African giraffe along a cryptic rift valley

**DOI:** 10.1186/s12862-014-0219-7

**Published:** 2014-10-23

**Authors:** Friederike Bock, Julian Fennessy, Tobias Bidon, Andy Tutchings, Andri Marais, Francois Deacon, Axel Janke

**Affiliations:** Biodiversity and Climate Research Centre (BiK-F) – Ecological Genomics & Senckenberg Gesellschaft für Naturforschung (SGN), Senckenberganlage 25, 60325 Frankfurt am Main, Germany; Giraffe Conservation Foundation, 26 Grasmere Road, Purley, Surrey, CR8 1DU England; School of Biological Earth and Environmental Studies (BEES), University of New South Wales (UNSW), Sydney, New South Wales 2052 Australia; Department Animal, Wildlife & Grassland Science, University of Free State, Faculty of Natural and Agricultural Sciences, Bloemfontein, South Africa; Goethe University Frankfurt, Institute for Ecology, Evolution & Diversity, Biologicum, Max-von-Laue-Straße 13, 60439 Frankfurt am Main, Germany

**Keywords:** *Giraffa*, Angolan giraffe, South African giraffe, Population genetics, Botswana, Namibia, Phylogeny, mtDNA

## Abstract

**Background:**

The current taxonomy of the African giraffe (*Giraffa camelopardalis*) is primarily based on pelage pattern and geographic distribution, and nine subspecies are currently recognized. Although genetic studies have been conducted, their resolution is low, mainly due to limited sampling. Detailed knowledge about the genetic variation and phylogeography of the South African giraffe (*G. c. giraffa*) and the Angolan giraffe (*G. c. angolensis*) is lacking. We investigate genetic variation among giraffe matrilines by increased sampling, with a focus on giraffe key areas in southern Africa.

**Results:**

The 1,562 nucleotides long mitochondrial DNA dataset (cytochrome b and partial control region) comprises 138 parsimony informative sites among 161 giraffe individuals from eight populations. We additionally included two okapis as an outgroup. The analyses of the maternally inherited sequences reveal a deep divergence between northern and southern giraffe populations in Africa, and a general pattern of distinct matrilineal clades corresponding to their geographic distribution. Divergence time estimates among giraffe populations place the deepest splits at several hundred thousand years ago.

**Conclusions:**

Our increased sampling in southern Africa suggests that the distribution ranges of the Angolan and South African giraffe need to be redefined. Knowledge about the phylogeography and genetic variation of these two maternal lineages is crucial for the development of appropriate management strategies.

**Electronic supplementary material:**

The online version of this article (doi:10.1186/s12862-014-0219-7) contains supplementary material, which is available to authorized users.

## Background

For more than 250 years, giraffe (*Giraffa camelopardalis*) taxonomy has attracted interest among scientists [1-3]. The descriptions of the nine giraffe subspecies are primarily based on pelage patterns, characteristics of ossicones and their geographic distribution across the African continent [4,5]. However, the inconsistent pelage recognition has confused taxonomical assignments due to its high variability [6-8]. Recent efforts using molecular genetic techniques are beginning to clarify giraffe taxonomy [9-11]. In contrast to studies on elephant [12,13], and other African wildlife [14,15], a range-wide genetic analysis of giraffe is lacking [9-11]. A phylogenetic study using data of six subspecies (Angolan giraffe (*G. c. angolensis*), South African giraffe (*G. c. giraffa*), West African giraffe (*G. c. peralta*), reticulated giraffe (*G. c. reticulata*), Rothschild’s giraffe (*G. c. rothschildi*) and Masai giraffe (*G. c. tippelskirchi*)) based on nuclear microsatellites and mitochondrial (mt) DNA sequences suggested that some of the subspecies may actually represent distinct species [9]. Another study of the giraffe subspecies historically classified as Thornicroft’s giraffe (*G. c. thornicrofti*), which is restricted to Zambia’s South Luangwa valley, showed that this population has a distinct mtDNA haplotype that is nested within the clade of Masai giraffe [11]. Genetic analysis suggested that the Kordofan giraffe (*G. c. antiquorum*) in Central Africa is closely related to the West African giraffe [10], while the relationship of the Nubian giraffe (*G. c. camelopardalis*) is unclear due to a lack of any genetic analyses.

In southern Africa, two subspecies of giraffe live in close proximity. South African giraffe have been reported to occur naturally throughout southern Botswana, southern Zimbabwe, southwestern Mozambique, northern South Africa and southeastern Namibia [7]. Giraffe of northwestern and north-central Namibia have been categorized as Angolan giraffe [1,16] but the taxonomic classification of giraffe from northern Botswana and northeastern Namibia remains uncertain. Angolan giraffe is thought to occur also in southern Zambia, western Zimbabwe and central Botswana [16]. Both giraffe populations have historically been classified as either *G. c. giraffa* or *G. c. angolensis*, or most recently as a hybrid of *G. giraffa*/*G. angolensis*, depending on the taxonomic reference [6,8]. The uncertainty of giraffe taxonomy in southern Africa affects conservation efforts, as individuals are being translocated both within and between different populations and countries across Africa without knowledge of the taxonomical status. Frequently, these translocations are driven by economic reasons for improving regional tourism rather than biodiversity conservation [17]. Conservation policies depend on reliable information about the taxonomic status and about genetic variability of locally adapted populations. Clarifying the relationship and distribution of the Angolan and South African giraffe is therefore particularly relevant for conservation efforts of the newly established Kavango-Zambezi Transfrontier Conservation Area (KAZA) that includes northeastern Namibia and northern Botswana.

Although no targeted census of giraffe has been conducted, the size of Botswana’s northern giraffe population is estimated to have dropped over the last decade from >10,000 to <4,000 individuals [18]. The number of giraffe in Bwabwata National Park in Namibia was decimated in the 1970s and 1980s due to illegal hunting but has recovered since then to >150 individuals [19].

We here present a population genetic analysis of mitochondrial cytochrome b (cytb) and partial control region (CR) sequences for eight of the nine currently described giraffe subspecies. Our sampling focuses on geographic regions that have not been analyzed before, particularly in southern Africa: Namibia (Bwabwata National Park – BNP, Etosha National Park – ENP) and Botswana (Chobe National Park – CNP, Moremi Game Reserve – MGR, Nxai Pans – NXP, Vumbura Concession – V, Central Kalahari Game Reserve – CKGR), but also central Africa’s Democratic Republic of Congo (Garamba National Park – GNP) (Table 1, Additional file 1: Table S1). Our dense sampling includes many key areas of the giraffe distribution range in southern Africa and therefore allows for a high-resolution analysis of the phylogeography of South African and Angolan giraffe. Furthermore, it allows assessing the impact of a “cryptic” rift valley, which runs northeast to southwest across Botswana from Zambia [20,21], potentially having acted as a barrier to giraffe dispersal.

**Table 1:** **Origin**, **abbreviation**, **number of individuals** (**N**) **and subspecies designation of analyzed giraffe sequences**

**Geographic origin**	**Abbreviation**	**N**	**Previous subspecies designation**	**Subspecies designation** **(this study)**
Vumbura Concession, Botswana	V	11	*angolensis*	*giraffa*
Chobe National Park, Botswana	CNP	11	*angolensis*	*giraffa*
Bwabwata National Park, Namibia	BNP	7	*angolensis*	*giraffa*
Moremi Game Reserve, Botswana	MGR	16	*angolensis*	*giraffa*
Nxai Pans, Botswana	NXP	1	*angolensis*	*giraffa*
Garamba National Park, DR Congo	GNP	3	*antiquorum*	*antiquorum*
Zakouma National Park, Chad	ZNP	1	*antiquorum*	*antiquorum*
Central Kalahari Game Reserve, Botswana	CKGR	7	*angolensis*	*angolensis*
Etosha National Park, Namibia	ENP	17	*angolensis*	*angolensis*
Khamab Kalahari Reserve, South Africa	KKR	6	*giraffa*	*giraffa*
Niger	WA	13	*peralta*	*peralta*
Murchison Falls National Park, Uganda	MF	9	*rothschildi*	*rothschildi*
Luangwa Valley, Zambia	LVNP	5	*thornicrofti*	*tippelskirchi*
Selous Game Reserve, Tanzania	SGR	6	*tippelskirchi*	*tippelskirchi*

## Results

Mitochondrial DNA sequences from the cytochrome b (cytb) gene and partial control region (CR) were successfully amplified from all samples. The cytb alignment was 1,140 nucleotides (nt) long and showed no gaps or ambiguous sites. We also sequenced the L-strand of the CR for a length of 786/787 nt, excluding the highly repetitive poly-cytosine region. In order to match our newly obtained sequences with published data, the length of the CR alignment was limited to 422 nt. The stringent 422 nt CR alignment did not contain gaps. The CR was relatively conserved outside this 422 nt region and until the poly-cytosine sequence, yielding only three variable sites among 20 giraffe individuals that represented all clades. All sequences conformed to the reading frame, length, stop codon and other properties of a functional protein coding gene or the control region that are observed in an established mitochondrial genome [EMBL: NC012100]. Sequences with the same properties were also obtained using the alternative primer pair for amplification and sequencing. Thus, it is reasonable to assume that no mitochondrial nuclear mitochondrial insertions (numts) were sequenced. The inclusion of two okapi (*Okapia johnstoni*) sequences introduced unambiguously placed gaps in the CR alignment, which were ignored in all subsequent analyses. The combined (cytb plus CR) alignment was 1,562 nt long and contained 138 parsimony informative sites. The alignment included 161 giraffe and two okapi individuals, of which 102 giraffe were newly sampled (Table 1, Additional file 1: Table S1).

**Figure 1:**
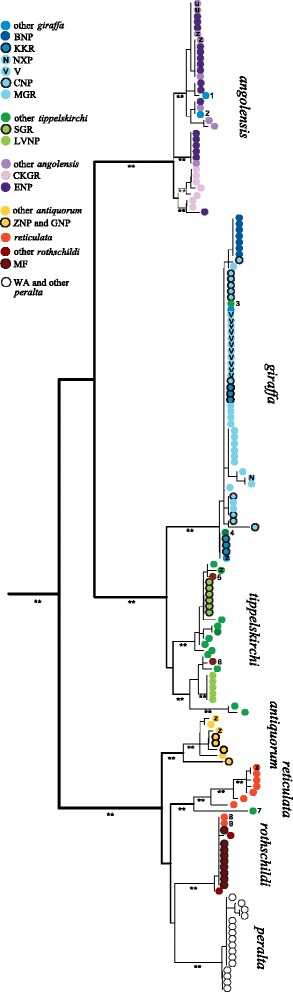
**Phylogenetic tree based on mitochondrial DNA encompassing 161 giraffe individuals.** The topology corresponds to a maximum clade credibility tree obtained from BEAST, but branch lengths were calculated by maximum likelihood in Treefinder. Each dot represents one individual giraffe, colors are coding for the respective subspecies/population. “z” denotes captive (zoo) individuals, asterisks at branches indicate Bayesian posterior support >0.95. Abbreviations for the samples are explained in the text and in Table 1.

The Bayesian analysis of mitochondrial sequence data recovered the matrilines of all giraffe subspecies to be monophyletic with respect to each other, although not all nodes received posterior support values above 0.95 (Figure 1). The most obvious pattern is a well-supported north-south split, with the southern subspecies Angolan giraffe, South African giraffe, and Masai/Thornicroft’s giraffe being separated from the northern subspecies Kordofan giraffe, reticulated giraffe, Rothschild’s giraffe and West African giraffe.

**Figure 2:**
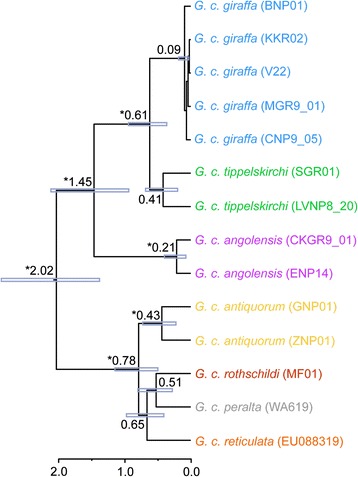
**Maximum clade credibility tree of the major giraffe populations as reconstructed by Bayesian analysis conducted in BEAST.** Blue bars indicate 95% highest posterior density intervals for node ages, asterisks denote posterior probability >0.95. Scale on the bottom represents divergence time (million years ago).

Using a molecular clock, BEAST estimates the deepest divergence time among giraffe matrilines between the northern and southern clade at ca. 2.0 million years ago (Ma) (Figure 2). This is followed by the divergence of a mtDNA clade containing Angolan giraffe, South African giraffe and Masai/Thornicroft’s giraffe at ca. 1.4 Ma (Table 2, Figure 2). A northern giraffe clade, which includes the Kordofan giraffe, reticulated giraffe, Rothschild’s giraffe, and West African giraffe, diverged at about 0.8 Ma (Table 2). Divergences within each subspecies are estimated to have occurred between 100 to 400 thousand years (ka) ago. Note that the Bayesian posterior support values for some of the nodes at the subspecies level were below 0.95 (Figure 2).

**Table 2:** **Divergence time estimates** (**median heights and 95**% **highest posterior density intervals**) **obtained from BEAST based on 1**,**565 nt mtDNA**

**Divergence**	**Time estimate** **(Ma)**
*G. c. giraffa* plus *tippelskirchi* plus *angolensis* vs. *antiquorum* plus *rothschildi* plus *peralta* plus *reticulata*	2.0 (1.4 - 2.8)*
*G. c. giraffa* plus *tippelskirchi* vs. *angolensis*	1.4 (0.9 - 2.1)*
*G. c. giraffa* vs. *tippelskirchi*	0.6 (0.4 - 0.9)*
*G. c. giraffa*	0.1 (0.02 - 0.2)
*G. c. tippelskirchi*	0.4 (0.2 - 0.7)
*G. c. angolensis*	0.2 (0.1 - 0.4)*
*G. c. antiquorum* vs. *rothschildi* plus *peralta* plus *reticulata*	0.8 (0.5 - 1.1)*
*G. c. rothschildi* plus *peralta* vs. *reticulata*	0.7 (0.4 - 1.0)
*G. c. rothschildi* vs. *peralta*	0.5 (0.3 - 0.8)
*G. c. antiquorum*	0.4 (0.2 - 0.7)*

Asterisks indicate posterior probability >0.95.

Giraffe from Luangwa Valley National Park, Zambia, which are formally classified as Thornicroft’s giraffe, form a uniform but not a separated matrilineal group within the variation of Masai giraffe. Note, that the divergence between the southern and northern clade occurs between populations south and north of the equator that are in close geographic proximity to each other (Masai giraffe, reticulated giraffe, Rothschild’s giraffe). The relative clustering of the northern mtDNA clades (West African giraffe, Rothschild’s giraffe, Kordofan giraffe and reticulated giraffe) remains uncertain due to low posterior support values for some of the nodes (Figure 1, Figure 2).

Nine database individuals that were assigned to a particular subspecies previously [9] grouped at unexpected positions in our phylogenetic analysis (numbered individuals in Figure 1). Two individuals of South African giraffe (# 1 and 2) are placed within Angolan giraffe but not with other South African giraffe individuals. Likewise, two individuals (# 3 and 4) of Masai giraffe are placed within South African giraffe, two Rothschild’s giraffe individuals (# 5 and 6) grouped with Masai giraffe, one Masai giraffe (# 7) fell basal to reticulated giraffe, and two reticulated giraffe (# 8 and 9) grouped with Rothschild’s giraffe. Additional information of the geographic origin of each individual sequence is given in Additional file 1: Table S1.

Currently, there are four giraffe subspecies recognized south of the equator in Africa: Masai/Thornicroft’s giraffe, South African giraffe, and Angolan giraffe, the two latter occurring in close proximity in Botswana. In our data, Angolan giraffe individuals from the Central Kalahari Game Reserve in central Botswana grouped with Angolan giraffe from the Etosha National Park in Namibia, which was expected from their geographic origin and previously assumed classification. One individual from the Etosha National Park fell into the Central Kalahari Game Reserve mtDNA clade.

**Figure 3:**
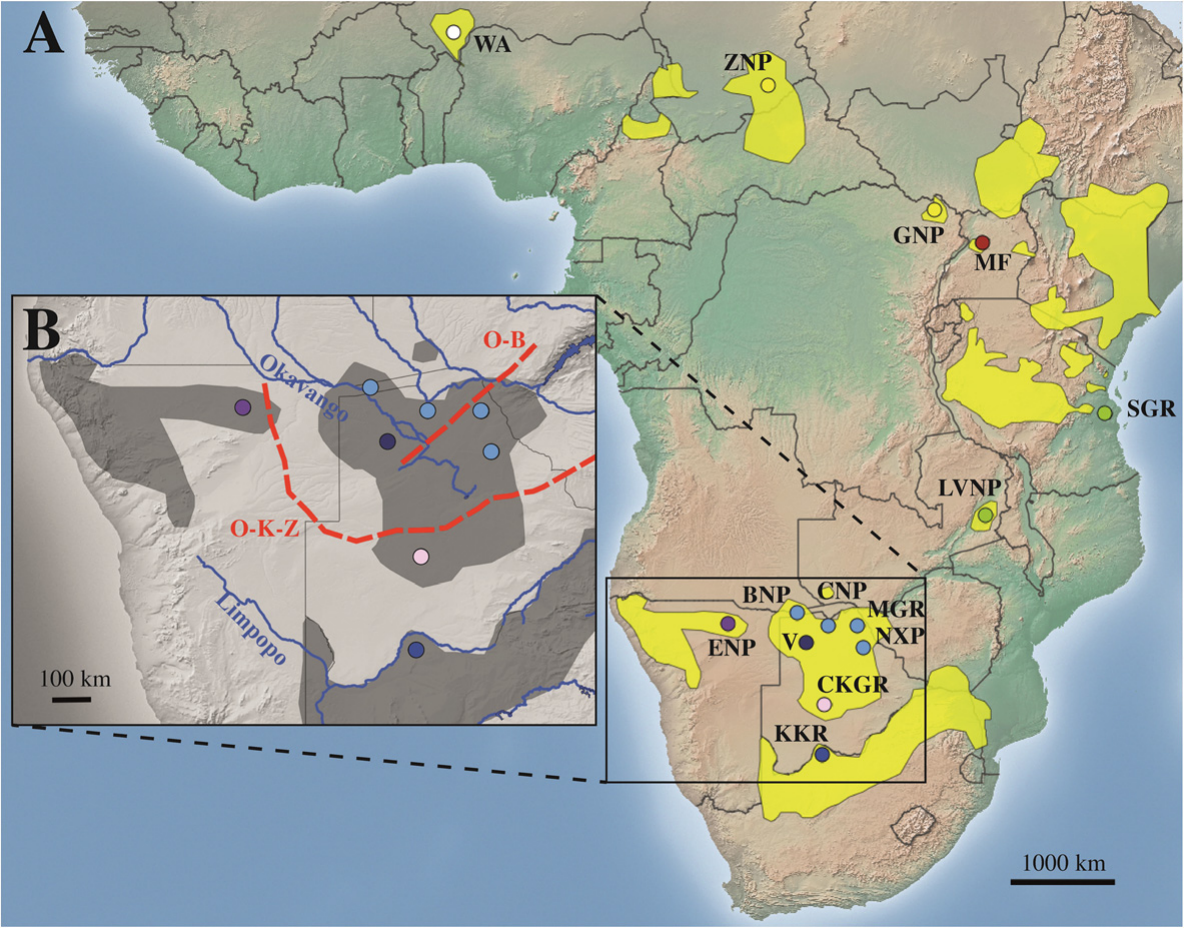
**Map of sub-Saharan Africa.** **A**: Distribution range of giraffe (yellow patches) and sampling locations (abbreviations are explained in Table 1). Colors show genetically identified subspecies (coding as in Figure 1). **B**: Depiction of southern African giraffe populations and location of geographic boundaries. O-K-Z: Owambo-Kalahari-Zimbabwe epigeiric axis, O-B: Okavango-Bangweulu axis.

Unexpectedly, 46 individuals sampled as Angolan giraffe from Chobe National Park, Nxai Pans, Vumbura Concession and Moremi Game Reserve in northern Botswana, and Bwabwata National Park in northeastern Namibia grouped with South African giraffe from the Khamab Kalahari Reserve in South Africa. These hitherto not sampled regions thus harbor mtDNA lineages of the South African giraffe subspecies and not of Angolan giraffe. Populations carrying the mitochondrial haplotype of South African giraffe thus geographically enclose the Angolan giraffe of the Central Kalahari Game Reserve from the north and south (Figure 3).

**Figure 4:**
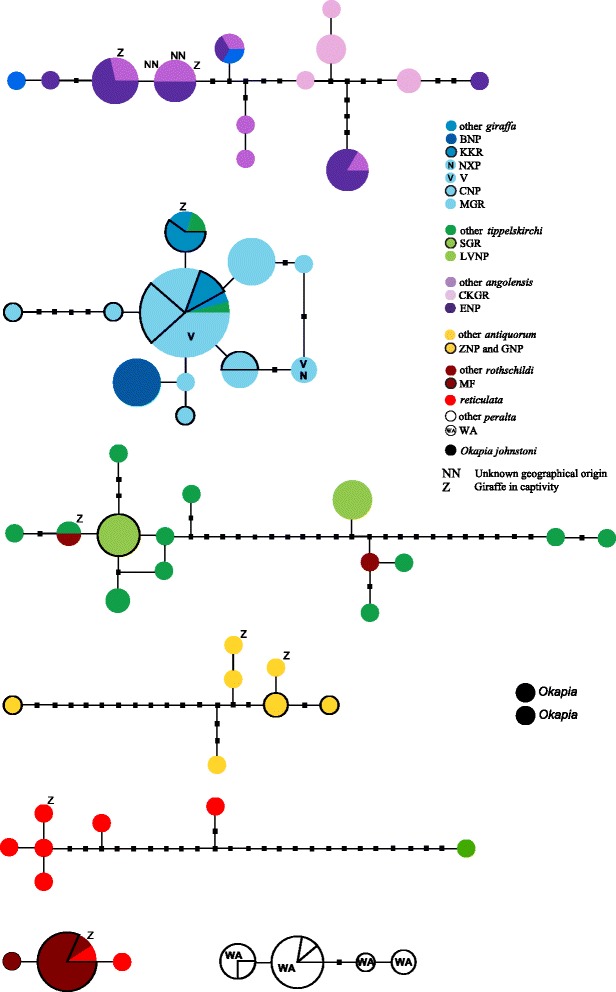
**Statistical parsimony haplotype network of the giraffe and okapi sequences.** The sub-networks of different giraffe subspecies do not connect at the 95% connection probability limit. Different populations having identical haplotypes are indicated by pie-sections. Black rectangles indicate not sampled haplotypes. Abbreviations as in Table 1.

Individuals from Bwabwata National Park formed a separate group with its own mtDNA haplotype (Figure 1, Figure 4).

To assess differentiation between populations, pairwise F_ST_ values were calculated (Table 3). The overall population differentiation of mtDNA was high, with F_ST_ values ranging from 0.672 (Masai giraffe and Thornicroft’s giraffe) to 0.998 (Rothschild’s giraffe and Thornicroft’s giraffe). The pairwise F_ST_ value between South African and Angolan giraffe was 0.929, showing a clear differentiation between those two populations, despite their close geographic proximity.

**Table 3:** **Genetic differentiation** (**pairwise F**_**ST**_ **values**) **among the eight subspecies as defined by mtDNA clades**

	***angolensis***	***giraffa***	***peralta***	***antiquorum***	***thornicrofti***	***tippelskirchi***	***reticulata***
*giraffa*	0.940						
*peralta*	0.935	0.978					
*antiquorum*	0.905	0.967	0.859				
*thornicrofti*	0.897	0.935	0.984	0.930			
*tippelskirchi*	0.859	0.838	0.916	0.866	0.506		
*reticulata*	0.901	0.961	0.839	0.689	0.900	0.856	
*rothschildi*	0.938	0.980	0.959	0.887	0.996	0.918	0.842

For assignment to mtDNA clades see Figure 1. All pairwise F_ST_ values were highly significant (p < 0.001) when testing with 1,000 permutations.

A haplotype network analysis supports the strong divergences among most giraffe mtDNA clades (Figure 4), as sub-networks representing the different subspecies are not connected to each other at the 95% connection probability limit. Corresponding to our phylogenetic analysis (Figure 1), Thornicroft’s giraffe are an exception, as individuals from the Luangwa valley share a distinct haplotype that falls within the variation of Masai giraffe. The networks also demonstrate the considerable amount of variation within most subspecies: Masai/Thornicroft’s and Angolan giraffe have the highest numbers of haplotypes (14 and 13, respectively; Table 4). Kordofan and reticulated giraffe show the highest haplotype diversities, 0.964 ± 0.077 and 0.972 ± 0.064, respectively – almost every individual has its own mitochondrial haplotype. In contrast, Thornicroft’s, West African and Rothschild’s giraffe have the lowest number of haplotypes, the lowest haplotype diversity, and the lowest nucleotide diversity (Table 4), corresponding to the short branch lengths of these three mtDNA clades (Figure 1). Although the overall mitochondrial variation in South African giraffe was comparable to that of other clades (13 haplotypes, H_d_ =0.769 ± 0.050; Table 4), it is noteworthy that one haplotype was common and shared among individuals from different reserves or parks (Vumbura Concession, Chobe National Park, Moremi Game Reserve, Nxai Pans, all in Botswana) (Figure 4).

**Table 4:** **Diversity indices per subspecies for the mtDNA**

	**N**	**N**_**H**_	**H**_**d**_	**sd** **(H**_**d**_ **)**	**π**	**sd** **(π)**
*angolensis*	33 (35)	13 (13)	0.902 (0.901)	0.028 (0.026)	0.00351 (0.00344)	0.00026 (0.00026)
*antiquorum*	8	**7**	0.964	0.077	0.00434	0.00140
*giraffa*	56 (56)	13 (11)	0.769 (0.751)	0.050 (0.052)	0.00326 (0.00103)	0.00147 (0.00017)
*reticulata*	9 (8)	8 (7)	0.972 (0.964)	0.064 (0.077)	0.00800 (0.00632)	0.00209 (0.00229)
*rothschildi*	13 (13)	4 (3)	0.423 (0.295)	0.164 (0.156)	0.01171 (0.00020)	0.00589 (0.00011)
*thornicrofti*	5	1	0.000	0.000	0.00000	0.00000
*tippelskirchi*	21 (20)	15 (13)	0.924 (0.911)	0.050 (0.054)	0.01030 (0.00555)	0.00319 (0.00119)
*peralta*	16	4	0.642	0.103	0.00082	0.00022
**Total**	**161**	**59**	**0.956**	**0.008**	**0.02667**	**0.00075**

N: number of analyzed individuals. N_H_: number of haplotypes. H_d_: haplotype diversity. sd: standard deviation. π: uncorrected nucleotide diversity. All indices were calculated in DnaSP. For previously published sequences, the original subspecies assignments were used. Our own samples are assigned to subspecies according to their mtDNA clades in Figure 1. Numbers in brackets are the respective indices when the probably misassigned individuals #1 to #9 are put in the mtDNA clades as presented in Figure 1.

Rothschild’s giraffe, which is currently considered “endangered” on the IUCN Red List [22], has two haplotypes among 11 individuals and low nucleotide and haplotype diversity (0.00012 ± 0.00009 and 0.182 ± 0.144, respectively; Table 4).

## Discussion

The analyses of 1,562 nt of concatenated mitochondrial sequence data identified seven well-separated and reciprocally monophyletic giraffe clades. The deepest divergence, as estimated by a Bayesian BEAST analysis, was found between a northern clade, comprising West African, Kordofan, reticulated, and Rothschild’s giraffe, and a southern clade, comprising Angolan, South African, and Masai/Thornicroft’s giraffe, despite the close geographic proximity of populations of both clades in East Africa. Notably, Masai giraffe are geographically much closer to northern populations than to the southern African Angolan and South African giraffe. The matrilineal clades identified are largely congruent to previously named subspecies and reflect the geographic structure seen among giraffe.

The Thornicroft’s giraffe has been described to only occur in the Luangwa Valley National Park. Divergences between Thornicroft’s and Masai giraffe are shallow, which is why the former was proposed to be subsumed into the Masai giraffe’s clade [11]. These lineages are on discrete evolutionary trajectories, due to their geographic isolation. The shallow divergence might thus reflect retention of ancestral polymorphisms, rendering mtDNA a marker with limited diagnostic resolution [23,24]. However, the giraffe from Luangwa Valley National Park have a unique mitochondrial haplotype (Figure 4). This should be taken into account in giraffe conservation and management, in particular for ecological, spatial and behavioral aspects. A previously suggested placing of the South African giraffe within the variation of the Masai giraffe [9] could not be confirmed. Our mtDNA tree shows the same topology as found by Hassanin and colleagues [10].

Assignment of individual giraffe to the wrong subspecies is not unusual and could be explained by natural migration or human-induced translocation. It is noteworthy, however, that every single one of the newly sampled 102 individuals was associated with the expected subspecies. Therefore, our data do not indicate large-scale migration of females from one subspecies to another or confusion of populations by human-induced translocation of females. Our new sampling effort of 102 individuals from well-defined areas and populations, and the data analyses indicate that individuals previously assigned to a clade different from the individual’s designation [9] might be a result of mtDNA introgression, or of inadequate subspecies identification. This highlights the importance of accurate sample collection and identification.

From previous studies [2] and historical assumptions [6], it was expected that Botswana and Namibia contain Angolan giraffe, and that the South African giraffe occurs further south and east in South Africa and Zimbabwe [2,6,25]. However, our data suggest a narrow zone separating Central Kalahari Game Reserve in Botswana, which is inhabited only by Angolan giraffe, from Chobe National Park, Moremi Game Reserve, Nxai Pans Park, and Vumbura Concession in northern Botswana, which are inhabited by South African giraffe. The central and northwestern giraffe populations in Namibia have formerly been assigned to Angolan giraffe [1,16]. Based on our results, the Bwabwata National Park population in northeastern Namibia unambiguously represents South African giraffe. The Bwabwata National Park population is geographically close (<100 km) to Chobe National Park and Vumbura Concession (also inhabited by South African giraffe), whereas the nearest natural Angolan giraffe population is >500 km to the west (Etosha National Park) or >350 km to the south (Central Kalahari Game Reserve).

Pairwise F_ST_ values of mtDNA sequences are expected to exceed those from nuclear markers in cases of strong female philopatry and male-biased gene flow or temporal nonequilibrium after a (recent) habitat fragmentation. In that case, mtDNA gene trees would show reciprocal monophyly and geographic structuring (as seen here), but nuclear loci would not support this [26].

The oldest fossils show that the giraffe species complex existed already about one Ma [27]. According to our divergence time estimates (Table 2, Figure 2), giraffe diverged into distinct populations that are designated as subspecies during the Pleistocene (2.6 Ma to 12 ka). This is considerably older than divergence times between closely related species of *Ursus* (~600 ka) estimated by independently inherited nuclear introns [28], of *Pan* (~420 ka) using multilocus analysis including mitochondrial, nuclear, X- and Y-chromosomal loci [29], or of *Canis* (~900 ka) based on mitochondrial genes and nuclear loci [30]. Due to the lack of sequence data from giraffe fossils and closely related and dated outgroup fossils, our calibration points (5 and 9 Ma, respectively) might lead to an overestimation of divergence times within giraffe. However, the clear intraspecific structuring into region-specific maternal clades supports an early divergence within giraffe. However, the mitochondrial gene tree might differ from the species tree [31], and a multilocus approach will be necessary to estimate divergence times representative of the species as a whole. Support for the early divergence time estimates comes from haplotype networks showing that numerous substitutions accumulated between matrilineal clades preventing connection at the 95% probability limit (Figure 4). Furthermore, there is considerable variation within most giraffe subspecies that can only develop during considerable time periods. Finally, signs of haplotype sharing between subspecies are rare (Figure 4), suggesting that maternal clades have been separated from each other for a considerable amount of time and that female gene flow among those clades is limited. However, it is not clear if the nine deviating individuals are misidentified samples, or if they result from human translocation or introgression of mtDNA among different giraffe populations. From 26 Masai/Thornicroft’s giraffe individuals, two share mtDNA haplotypes with South African giraffe, and one has a unique haplotype similar to reticulated giraffe (Figure 1, Figure 4). Evidence from autosomal microsatellites supports the clear structuring into subspecific groups, although limited signs of allele sharing were found among some populations [9].

Today, the majority of giraffe populations analyzed are widely separated and geographically isolated. This is a consequence of increasing agricultural practices causing habitat loss and fragmentation, of human population and settlement growth, and illegal hunting. Historically, and during the Pleistocene, the distribution ranges may have been more contiguous. Yet, during the Pleistocene, some barriers must have limited female gene flow among different giraffe populations. The distribution of many African ungulates is correlated closely with the distribution of savannah habitat, which in turn is strongly influenced by climatic conditions. The African climate experienced wide changes during the Pleistocene, resulting in recurrent expansions and contractions of savannah habitat and tropical forest. An increase of tropical forest across Central Africa during warm and wet periods (pluvials) around the equator might explain the north-south split seen today in giraffe and other ungulates [32,33]. In the northern parts of the distribution range, the expansion of the Lake Mega-Chad at about 8,000 to 3,000 years ago [34], might have affected recent giraffe dispersal [10].

We dated the divergence between the Angolan and South African giraffe matrilines in Botswana to 1.4 Ma. This deep, early Pleistocene divergence exists despite their close geographic proximity: distances up to 300 km can be travelled by giraffe [35]. Today, no obvious geographic barrier appears to separate these two subspecies. Thus, we propose a historical “cryptic” rift valley as explanation for the pattern seen in Botswana, as outlined below.

A known geographic boundary follows the Okavango River (Figure 3B) and Gumare Fault in the northwest of Botswana and extends east to the Thamalakhane Fault south of the Okavango pans and the Ntwetwe Pan. The Owambo-Kalahari-Zimbabwe epeirogenic axis (O-K-Z; Figure 3B) also forms a subtle but yet distinct geographic boundary [21,20] between Angolan and South African giraffe populations. Today, this area only holds seasonal water and thus does not seem as an obvious barrier to dispersal. However, it could have been a barrier during the Pleistocene [21,36]. The Okavango-Bangweulu axis (O-B; Figure 3B) is the southern extension of the East African Rift System and could have acted as further geographic separator when mountains were lowered and drainage systems formed resulting in the north-east split of giraffe matrilines. The persistence of these conditions might have been reinforced, if an early Pleistocene interglacial coincided with a maximum extent of Palaeo-Lake Makgadikgadi, which ended likely before the Middle Pleistocene (~970 to 500 ka) [21,36]. It has been suggested that a “cryptic” rift valley runs northeast to southwest across Botswana from Zambia with faulting ramifying southwest which is represented best by the development of the Fish River canyon in southern Namibia [37]. There were massive lake systems in northeast Botswana, but these dwindled by 500 to 600 ka (Palaeo-Lake Thamalakhane) [21]. Cotterill [36] argues that the above described phylogeographic anomaly is a result of an expansion of moist, evergreen forests in an interglacial, e. g. during warm and wet conditions. Such a “cryptic” rift valley can also explain distributions of other animals that are similar to the distribution of giraffe mtDNA haplotypes: African forest elephant (*Loxodonta cyclotis*) haplotypes are not within the variation of the African elephant (*L. africana*) from central Namibia (and southeast Botswana), but are confined only to the populations in northern Botswana and northwestern Zambia [12]. Phylogeographic divergences between southeast and northeast representatives of the Damara dikdik (*Madoqua damarensis*) and the impala (*Aepyceros petersi*) [38] exhibit both congruent distributions with Angolan giraffe in Namibia, as a result of Pleistocene climatic conditions and/or major changes in the larger rivers on the south-central African plateau during the Pleistocene [39]. Finally, the estimated population expansion of the Okavango Red lechwes (*Kobus leche*), a floodplain specialist, is explained by expansion of floodplain habitats following contraction of the northeast Botswana mega-lakes in the Middle Pleistocene [36].

Thus, the persistence of a vast mosaic of aquatic habitats and moist forest occupied the shallow rift valley of northeast Botswana through much of the Pleistocene [21]. This scenario poses a conceivable explanation for the formation of the distribution of Angolan and South African giraffe maternal lineages as currently seen in Botswana. Today, no obvious geographic barrier appears to separate these two subspecies. Ecological or behavioral factors, such as a specific mate recognition system [40], possibly differentiated pelage pattern and female philopatry may maintain limited genetic admixture.

A major episode of aridity in a Pleistocene glacial period may explain mtDNA lineage divergence within Angolan giraffe populations being restricted to Namibia (including Etosha National Park), and one being located in central Botswana (Central Kalahari Game Reserve). Few large mammals show such phylogeographic evidence of strong influence by geological landforms in the form of genetic depauperation or change in the extant distributions across southern Angola, northeastern Botswana and southwestern Zambia [39].

Mitochondrial DNA is maternally inherited from mother to offspring. It allows tracing the maternal lineage and reflects female movements, or the lack thereof, in a phylogeographic context. While we acknowledge the pitfalls of only investigating a small, uniparentally inherited part of the genome [26], mtDNA nevertheless enabled us to specifically analyze the maternal lineages of giraffe subspecies and also include database sequences of reticulated giraffe, for which samples are lacking. Reticulated giraffe are interesting due to their high variability and close proximity to subspecies of the southern clade. Moreover, it has been shown previously that phylogenetic trees based on mtDNA and nuclear microsatellites are congruent in giraffe [9], suggesting that the matrilineal structuring is not differing considerably from that of the species as a whole. The clear structure of the mtDNA clades might thus allow inferring that giraffe populations (and not only the matrilines) have been separated from each other for a considerable amount of time. Alternatively, mtDNA structure might reflect the nature of females to stay at or return to their place of birth (philopatry or site fidelity). Although female philopatry and male-biased dispersal has not been systematically studied in wild giraffe, it is a general pattern in many mammals [41]. However, long-term field observations by one of the authors (JF) support fidelity of both sexes of giraffe to a particular region, because the populations of desert-dwelling Angolan giraffe in northwest Namibia remained without contact and genetic admixture for at least five years, despite close proximity to other giraffe in Etosha National Park approximately 150 to 200 km east. The effects of male-biased gene flow on phylogeographic structuring of a widely distributed species have recently been demonstrated in bears [42]. To further investigate if giraffe represent one species with matrilineal structuring or a multi-species complex, and to analyze the extent of mitochondrial and nuclear discordance [43], future research must incorporate multiple independently inherited autosomal loci. The differences in pelage pattern observed among giraffe from different regions might reflect nuclear variation, indicative of separation between subspecies also at biparentally inherited parts of the genome. Moreover, markers from the paternally inherited Y chromosome would be beneficial to specifically study male gene flow to recover a potentially contrasting structuring of the patriline. If giraffe exhibited male-biased dispersal and if several species were involved, female-specific mtDNA is predicted to be a marker with high introgression rates, showing insufficiently diagnostic resolution on species delimitation [23].

## Conclusions

Enhanced sampling from key regions of the giraffe distribution range show a clear matrilineal structuring of giraffe into distinct clades. The genetic analyses support a clear north-south split, separating two major matrilineal clades in giraffe (southern and northern clade). We also found a sharp east-west delineation between Angolan and South African giraffe, in an area in northern Botswana that has not been genetically investigated before. Our study shows for the first time that South African giraffe are distributed in different parks in Botswana, north of their previously known distribution range. Biparentally and/or paternally inherited sequence markers will be the next step to fully understand the subspecies/species structure in this wide-spread charismatic African mammal.

## Methods

We collected giraffe tissue samples from seven of nine currently described subspecies (Table 1) (*G. c. angolensis*, *G. c. giraffa*, *G. c. tippelskirchi*, *G. c. antiquorum*, *G. c. rothschildi*, *G. c. peralta*, *G. c. thornicrofti*) and included published data for *G. c. reticulata* (Additional file 1: Table S1) in our analyses. In August 2009, samples for seven subspecies were collected using remote delivery biopsy darting from free-ranging giraffe in major giraffe populations in northern and central Botswana: Moremi Game Reserve (MGR), Chobe National Park (CNP), Central Kalahari Game Reserve (CKGR) and Nxai Pans (NXP). In 2013, samples were collected from the Vumbura Concession (V) and northern Okavango Delta in Botswana, and from Bwabwata National Park (BNP) in northeastern Namibia (Figure 3). Additional samples were collected in collaboration with conservation partners in Chad, Democratic Republic of Congo, Niger, South Africa, Tanzania and Uganda (Table 1, Additional file 1: Table S1). Skin biopsies were stored at room temperature in a tissue preservative buffer [44] with glutaraldehyde prior to DNA isolation. Whole genomic DNA was extracted from tissue and blood using standard phenol/chloroform extraction [45].

The complete cytb gene and a partial CR were PCR amplified and sequenced with newly designed giraffe-specific PCR primers that were constructed from an existing mitochondrial genome of the giraffe [EMBL AP003424]. The 1,140 nt long cytb gene was amplified with the primer pair 5’-TGAAAAACCATCGTTGTCGT-3’ and 5’-GTGGAAGGCGAAGAATCG-3’ and the control region (422 nt) was amplified with the primer pair 5’-TGAAAAACCATCGTTGTCGT-3’ and 5’-GTGGAAGGCGAAGAATCG-3’. In rare cases where amplification or sequencing produced unintelligible sequences or sequences with poor quality, mitochondrial-specific sequences were obtained with an alternative primer pair (5’-GACCCACCAAAATTTAACACAATC-3’ and 5’-GTATGAAGTCTGTGTTGGTCGTTG-3’).

PCR amplification of mtDNA sequences was performed with 10 ng genomic DNA using the VWR Mastermix containing Amplicon-Taq (VWR International GmbH, Darmstadt, Germany) according to the following protocol: 6 μL 2× mastermix incl. Taq, 0.25 μL 100× bovine serum albumin, 0.4 μL 10 pmol/μL each forward and reverse primer, 6.45 μL desalted water, DNA. PCR conditions for were as follows: initiation at 95°C for 5 min, 35 cycles of denaturation (at 95°C for 30 s), annealing (at 50°C for 30 s) and elongation (at 72°C for 1 min), and a final elongation step at 72°C for 5 min. The PCR products were diluted in water and cycle sequencing was done with the BigDye terminator sequencing kit 3.1 (Applied Biosystems, Foster City, California). Excess dye was removed with the BigDye XTerminator Purification Kit (Applied Biosystems). Purified products were analyzed on an Applied Biosystems ABI 3730 DNA Analyzer [EMBL: HG975087-HG975290].

Our data set was complemented with published sequences from databases (listed in Additional file 1: Table S1) e.g. from [9,10,46]. Sequences were manually edited in Geneious version 5.6.4 (Biomatters, Auckland, New Zealand) and aligned with ClustalX [47]. The corresponding sequences from two okapis (*Okapia johnstoni*) database samples [EMBL: JN632674, HF571214, HF571175] were used as outgroup.

TCS 1.21 [48] inferred statistical parsimony haplotype networks with the connection probability limit set to 95%. Columns containing ambiguous sites were removed from the alignment and gaps were treated as fifth state. DnaSP 5.10 [49] was used for the calculation of nucleotide diversity, number of haplotypes and haplotype diversity and Arlequin ver 3.5 [50] for pairwise F_ST_ values. Inkscape 0.48 was used to improve trees and networks graphics.

For divergence time estimations, mtDNA sequences from suitable ruminants (*Pudu puda*, *Rangifer tarandus*, *Muntiacus muntjak* and *Cervus elaphus*) were obtained from EMBL/GenBank (Additional file 1: Table S1). The split between *Pudu puda* and *Rangifer tarandus* was set to 5 Ma and between *Muntiacus muntjak* and *Cervus elaphus* to 9 Ma according to the fossil record [46]. A Bayesian phylogenetic tree including all 161 giraffe individuals and two okapis was estimated in BEAST v1.7.5 [51]. The branch length were calculated on the BEAST tree topology in TREEFINDER version of March 2008 using a maximum likelihood approach [52]. Coalescent based divergence times were estimated in BEAST on a restricted subset of the giraffe individuals in order to avoid an imbalance between taxon sampling of giraffe and outgroups. The subset included one representative of each subspecies and major population. We used the HKY + I + G substitution model as identified best fitting by jModelTest [53], a lognormal relaxed clock with a uniform prior on the substitution rate and ran the program for 2×10^8^ generations. Convergence was confirmed in Tracer v1.5.

### Availability of supporting data

DNA sequences are deposited at GenBank under the accession numbers [EMBL: HG975087-HG975290].

## Additional file

Additional file 1
**Sample information with locations, accession numbers, and subspecies designation.**

## Abbreviations

Cytb: Cytochrome b
CR: Control region
H_d_: Haplotype diversity
ka: Thousand years
KAZA: Kavango-Zambezi
Ma: Million years
mt: Mitochondrial
nt: Nucleotides
numts: Nuclear mitochondrial DNA
O-K-Z epigeiric axis: Owambo-Kalahari-Zimbabwe epigeiric axis
O-B axis: Okavango-Bangweulu axis
PCR: Polymerase chain reaction
