# Supplementary material for: Mitochondrial sequences reveal a clear separation between Angolan and South African giraffe along a cryptic rift valley
Source: BMC Evol Biol. 2014 Oct 23;14:219. doi: 10.1186/s12862-014-0219-7 (PMC4207324; doi:10.1186/s12862-014-0219-7)
Supplement: Additional file 1 — Sample information with locations, accession numbers, and subspecies designation. [file 12862_2014_219_MOESM1_ESM.docx]

**Supplementary table 1**

| **Scientific name (common name)** | **Geographical origin** | **Individual code** | **Sequence accession number** | **Reference** |
| --- | --- | --- | --- | --- |
| *G. c. angolensis* (Angolan giraffe) | Unknown | AP003424 | AP003424 | Yasue et al. 2001 unpublished |
| *G. c. giraffa* (South African giraffe) | Bwabwata National Park, Namibia | BNP01 | HG975087  HG975189 | Current paper |
| *G. c. giraffa* (South African giraffe) | Bwabwata National Park, Namibia | BNP04 | HG975088  HG975190 | Current paper |
| *G. c. giraffa* (South African giraffe) | Bwabwata National Park, Namibia | BNP05 | HG975089  HG975191 | Current paper |
| *G. c. giraffa* (South African giraffe) | Bwabwata National Park, Namibia | BNP06 | HG975090  HG975192 | Current paper |
| *G. c. giraffa* (South African giraffe) | Bwabwata National Park, Namibia | BNP07 | HG975091  HG975193 | Current paper |
| *G. c. giraffa* (South African giraffe) | Bwabwata National Park, Namibia | BNP08 | HG975092  HG975194 | Current paper |
| *G. c. giraffa* (South African giraffe) | Bwabwata National Park, Namibia | BNP09 | HG975093  HG975195 | Current paper |
| *G. c. angolensis* (Angolan giraffe) | Central Kalahari Game Reserve, Botswana | CKGR9_01 | HG975094  HG975196 | Current paper |
| *G. c. angolensis* (Angolan giraffe) | Central Kalahari Game Reserve, Botswana | CKGR9_02 | HG975095  HG975197 | Current paper |
| *G. c. angolensis* (Angolan giraffe) | Central Kalahari Game Reserve, Botswana | CKGR9_03 | HG975096  HG975198 | Current paper |
| *G. c. angolensis* (Angolan giraffe) | Central Kalahari Game Reserve, Botswana | CKGR9_05 | HG975097  HG975199 | Current paper |
| *G. c. angolensis* (Angolan giraffe) | Central Kalahari Game Reserve, Botswana | CKGR9_06 | HG975098  HG975200 | Current paper |
| *G. c. angolensis* (Angolan giraffe) | Central Kalahari Game Reserve, Botswana | CKGR9_07 | HG975099  HG975201 | Current paper |
| *G. c. angolensis* (Angolan giraffe) | Central Kalahari Game Reserve, Botswana | CKGR9_08 | HG975100  HG975202 | Current paper |
| *G. c. giraffa* (South African giraffe) | Chobe National Park, Botswana | CNP9_02 | HF571172  HF571215 | Fennessy et al. 2013 |
| *G. c. giraffa* (South African giraffe) | Chobe National Park, Botswana | CNP9_04 | HG975101  HG975203 | Current paper |
| *G. c. giraffa* (South African giraffe) | Chobe National Park, Botswana | CNP9_05 | HF571174  HF571217 | Fennessy et al. 2013 |
| *G. c. giraffa* (South African giraffe) | Chobe National Park, Botswana | CNP9_06 | HG975102  HG975204 | Current paper |
| *G. c. giraffa* (South African giraffe) | Chobe National Park, Botswana | CNP9_07 | HG975103  HG975205 | Current paper |
| *G. c. giraffa* (South African giraffe) | Chobe National Park, Botswana | CNP9_08 | HG975104  HG975206 | Current paper |
| *G. c. giraffa* (South African giraffe) | Chobe National Park, Botswana | CNP9_09 | HG975105  HG975207 | Current paper |
| *G. c. giraffa* (South African giraffe) | Chobe National Park, Botswana | CNP9_10 | HG975106  HG975208 | Current paper |
| *G. c. giraffa* (South African giraffe) | Chobe National Park, Botswana | CNP9_12 | HG975107  HG975209 | Current paper |
| *G. c. giraffa* (South African giraffe) | Chobe National Park, Botswana | CNP9_13 | HG975108  HG975210 | Current paper |
| *G. c. giraffa* (South African giraffe) | Chobe National Park, Botswana | CNP9_14 | HG975109  HG975211 | Current paper |
| *G. c. angolensis* (Angolan giraffe) | Lisbon Zoo | EF442263 | EF442263 | Hassanin et al. 2007 |
| *G. c. angolensis* (Angolan giraffe) | Lisbon Zoo | EF442264 | EF442264 | Hassanin et al. 2007 |
| *G. c. antiquorum* (Kordofan giraffe) | Vincennes Zoo | EF442265 | EF442265 | Hassanin et al. 2007 |
| *G. c. antiquorum* (Kordofan giraffe) | Chad | EF442266 | EF442266 | Hassanin et al. 2007 |
| *G. c. antiquorum* (Kordofan giraffe) | Antwerp Zoo | EF442267 | EF442267 | Hassanin et al. 2007 |
| *G. c. antiquorum* (Kordofan giraffe) | Cameroon | EF442268 | EF442268 | Hassanin et al. 2007 |
| *G. c. tippelskirchi* (Masai giraffe) | Basel Zoo | EF442269 | EF442269 | Hassanin et al. 2007 |
| *G. c. giraffa* (South African giraffe) | Thoiry Zoological Park | EF442270 | EF442270 | Hassanin et al. 2007 |
| *G. c. giraffa* (South African giraffe) | South Africa | EF442271 | EF442271 | Hassanin et al. 2007 |
| *G. c. reticulata* (Reticulated giraffe) | Sigean African Reserve | EF442272 | EF442272 | Hassanin et al. 2007 |
| *G. c. rothschildi* (Rothschild’s giraffe) | Thoiry Zoological Park | EF442273 | EF442273 | Hassanin et al. 2007 |
| *G. c. peralta* (West African giraffe) | Niger | EF442274 | EF442274 | Hassanin et al. 2007 |
| *G. c. angolensis* (Angolan giraffe) | Etosha National Park, Namibia | ENP01 | HG975110  HG975212 | Current paper |
| *G. c. angolensis* (Angolan giraffe) | Etosha National Park, Namibia | ENP04 | HG975111  HG975213 | Current paper |
| *G. c. angolensis* (Angolan giraffe) | Etosha National Park, Namibia | ENP05 | HG975112  HG975214 | Current paper |
| *G. c. angolensis* (Angolan giraffe) | Etosha National Park, Namibia | ENP06 | HG975113  HG975215 | Current paper |
| *G. c. angolensis* (Angolan giraffe) | Etosha National Park, Namibia | ENP07 | HG975114  HG975216 | Current paper |
| *G. c. angolensis* (Angolan giraffe) | Etosha National Park, Namibia | ENP08 | HG975115  HG975217 | Current paper |
| *G. c. angolensis* (Angolan giraffe) | Etosha National Park, Namibia | ENP11 | HG975116  HG975218 | Current paper |
| *G. c. angolensis* (Angolan giraffe) | Etosha National Park, Namibia | ENP12 | HG975117  HG975219 | Current paper |
| *G. c. angolensis* (Angolan giraffe) | Etosha National Park, Namibia | ENP13 | HG975118  HG975220 | Current paper |
| *G. c. angolensis* (Angolan giraffe) | Etosha National Park, Namibia | ENP14 | HG975119  HG975221 | Current paper |
| *G. c. angolensis* (Angolan giraffe) | Etosha National Park, Namibia | ENP15 | HG975120  HG975222 | Current paper |
| *G. c. angolensis* (Angolan giraffe) | Etosha National Park, Namibia | ENP16 | HG975121  HG975223 | Current paper |
| *G. c. angolensis* (Angolan giraffe) | Etosha National Park, Namibia | ENP17 | HG975122  HG975224 | Current paper |
| *G. c. angolensis* (Angolan giraffe) | Etosha National Park, Namibia | ENP18 | HG975123  HG975225 | Current paper |
| *G. c. angolensis* (Angolan giraffe) | Etosha National Park, Namibia | ENP19 | HG975124  HG975226 | Current paper |
| *G. c. angolensis* (Angolan giraffe) | Etosha National Park, Namibia | ENP20 | HG975125  HG975227 | Current paper |
| *G. c. angolensis* (Angolan giraffe) | Etosha National Park, Namibia | ENP21 | HG975126  HG975228 | Current paper |
| *G. c. peralta* (West African giraffe) | Niger | EU088317 | EU088317 | Brown et al. 2007 |
| *G. c. peralta* (West African giraffe) | Niger | EU088318 | EU088318 | Brown et al. 2007 |
| *G. c. reticulata* (Reticulated giraffe) | Kenya | EU088319 | EU088319 | Brown et al. 2007 |
| *G. c. reticulata* (Reticulated giraffe) | Kenya | EU088320 | EU088320 | Brown et al. 2007 |
| *G. c. reticulata* (Reticulated giraffe) | Kenya | EU088322 | EU088322 | Brown et al. 2007 |
| *G. c. reticulata* (Reticulated giraffe) | Kenya | EU088323 | EU088323 | Brown et al. 2007 |
| *G. c. reticulata* (Reticulated giraffe) | Kenya | EU088324 | EU088324 | Brown et al. 2007 |
| *G. c. reticulata* (Reticulated giraffe) | Kenya | EU088325 | EU088325 | Brown et al. 2007 |
| *G. c. reticulata* (Reticulated giraffe) | Kenya | EU088326 | EU088326 | Brown et al. 2007 |
| *G. c. reticulata* (Reticulated giraffe) | Kenya | EU088327 | EU088327 | Brown et al. 2007 |
| *G. c. rothschildi* (Rothschild’s giraffe) | Uganda or Kenya | EU088328 | EU088328 | Brown et al. 2007 |
| *G. c. rothschildi* (Rothschild’s giraffe) | Uganda or Kenya | EU088329 | EU088329 | Brown et al. 2007 |
| *G. c. rothschildi* (Rothschild’s giraffe) | Uganda or Kenya | EU088330 | EU088330 | Brown et al. 2007 |
| *G. c. tippelskirchi* (Masai giraffe) | Kenya or Tanzania | EU088331 | EU088331 | Brown et al. 2007 |
| *G. c. tippelskirchi* (Masai giraffe) | Kenya or Tanzania | EU088332 | EU088332 | Brown et al. 2007 |
| *G. c. tippelskirchi* (Masai giraffe) | Kenya or Tanzania | EU088333 | EU088333 | Brown et al. 2007 |
| *G. c. tippelskirchi* (Masai giraffe) | Kenya or Tanzania | EU088334 | EU088334 | Brown et al. 2007 |
| *G. c. tippelskirchi* (Masai giraffe) | Kenya or Tanzania | EU088335 | EU088335 | Brown et al. 2007 |
| *G. c. tippelskirchi* (Masai giraffe) | Kenya or Tanzania | EU088336 | EU088336 | Brown et al. 2007 |
| *G. c. tippelskirchi* (Masai giraffe) | Kenya or Tanzania | EU088337 | EU088337 | Brown et al. 2007 |
| *G. c. tippelskirchi* (Masai giraffe) | Kenya or Tanzania | EU088338 | EU088338 | Brown et al. 2007 |
| *G. c. tippelskirchi* (Masai giraffe) | Kenya or Tanzania | EU088339 | EU088339 | Brown et al. 2007 |
| *G. c. tippelskirchi* (Masai giraffe) | Kenya or Tanzania | EU088340 | EU088340 | Brown et al. 2007 |
| *G. c. tippelskirchi* (Masai giraffe) | Kenya or Tanzania | EU088341 | EU088341 | Brown et al. 2007 |
| *G. c. tippelskirchi* (Masai giraffe) | Kenya or Tanzania | EU088342 | EU088342 | Brown et al. 2007 |
| *G. c. tippelskirchi* (Masai giraffe) | Kenya or Tanzania | EU088343 | EU088343 | Brown et al. 2007 |
| *G. c. tippelskirchi* (Masai giraffe) | Kenya or Tanzania | EU088344 | EU088344 | Brown et al. 2007 |
| *G. c. giraffa* (South African giraffe) | South Africa or Zimbabwe | EU088345 | EU088345 | Brown et al. 2007 |
| *G. c. giraffa* (South African giraffe) | South Africa or Zimbabwe | EU088346 | EU088346 | Brown et al. 2007 |
| *G. c. angolensis* (Angolan giraffe) | Namibia | EU088347 | EU088347 | Brown et al. 2007 |
| *G. c. angolensis* (Angolan giraffe) | Namibia | EU088348 | EU088348 | Brown et al. 2007 |
| *G. c. angolensis* (Angolan giraffe) | Namibia | EU088349 | EU088349 | Brown et al. 2007 |
| *G. c. angolensis* (Angolan giraffe) | Namibia | EU088350 | EU088350 | Brown et al. 2007 |
| *G. c. angolensis* (Angolan giraffe) | Namibia | EU088351 | EU088351 | Brown et al. 2007 |
| *G. c. antiquorum* (Kordofan giraffe) | Garamba National Park, DR Congo | GNP01 | HG975127  HG975229 | Current paper |
| *G. c. antiquorum* (Kordofan giraffe) | Garamba National Park, DR Congo | GNP02 | HG975128  HG975230 | Current paper |
| *G. c. antiquorum* (Kordofan giraffe) | Garamba National Park, DR Congo | GNP04 | HG975129  HG975231 | Current paper |
| *Okapia johnstoni* | NN | JN632674 | JN632674 | Hassanin et al. 2012 |
| *G. c. tippelskirchi* (Masai giraffe) | Luangwa Valley, Zambia | LVNP8_20 | HF571148  HF571190 | Fennessy et al. 2013 |
| *G. c. tippelskirchi* (Masai giraffe) | Luangwa Valley, Zambia | LVNP8_21 | HF571149  HF571191 | Fennessy et al. 2013 |
| *G. c. tippelskirchi* (Masai giraffe) | Luangwa Valley, Zambia | LVNP8_22 | HF571150  HF571192 | Fennessy et al. 2013 |
| *G. c. tippelskirchi* (Masai giraffe) | Luangwa Valley, Zambia | LVNP8_25 | HF571151  HF571193 | Fennessy et al. 2013 |
| *G. c. tippelskirchi* (Masai giraffe) | Luangwa Valley, Zambia | LVNP8_41 | HF571166  HF571208 | Fennessy et al. 2013 |
| *G. c. rothschildi* (Rothschild’s giraffe) | Murchison Falls National Park,Uganda | MF01 | HG975130  HG975232 | Current paper |
| *G. c. rothschildi* (Rothschild’s giraffe) | Murchison Falls National Park,Uganda | MF02 | HG975131  HG975233 | Current paper |
| *G. c. rothschildi* (Rothschild’s giraffe) | Murchison Falls National Park,Uganda | MF03 | HG975132  HG975234 | Current paper |
| *G. c. rothschildi* (Rothschild’s giraffe) | Murchison Falls National Park,Uganda | MF04 | HG975133  HG975235 | Current paper |
| *G. c. rothschildi* (Rothschild’s giraffe) | Murchison Falls National Park,Uganda | MF05 | HG975134  HG975236 | Current paper |
| *G. c. rothschildi* (Rothschild’s giraffe) | Murchison Falls National Park,Uganda | MF06 | HG975135  HG975237 | Current paper |
| *G. c. rothschildi* (Rothschild’s giraffe) | Murchison Falls National Park,Uganda | MF07 | HG975136  HG975238 | Current paper |
| *G. c. rothschildi* (Rothschild’s giraffe) | Murchison Falls National Park,Uganda | MF08 | HG975137  HG975239 | Current paper |
| *G. c. rothschildi* (Rothschild’s giraffe) | Murchison Falls National Park,Uganda | MF09 | HG975138  HG975240 | Current paper |
| *G. c. giraffa* (South African giraffe) | Moremi Game Reserve, Botswana | MGR9_01 | HG975139  HG975241 | Current paper |
| *G. c. giraffa* (South African giraffe) | Moremi Game Reserve, Botswana | MGR9_02 | HG975140  HG975242 | Current paper |
| *G. c. giraffa* (South African giraffe) | Moremi Game Reserve, Botswana | MGR9_03 | HG975141  HG975243 | Current paper |
| *G. c. giraffa* (South African giraffe) | Moremi Game Reserve, Botswana | MGR9_04 | HF571168  HF571210 | Fennessy et al. 2013 |
| *G. c. giraffa* (South African giraffe) | Moremi Game Reserve, Botswana | MGR9_05 | HG975142  HG975244 | Current paper |
| *G. c. giraffa* (South African giraffe) | Moremi Game Reserve, Botswana | MGR9_06 | HG975143  HG975245 | Current paper |
| *G. c. giraffa* (South African giraffe) | Moremi Game Reserve, Botswana | MGR9_09 | HG975144  HG975246 | Current paper |
| *G. c. giraffa* (South African giraffe) | Moremi Game Reserve, Botswana | MGR9_11 | HF571169  HF571211 | Fennessy et al. 2013 |
| *G. c. giraffa* (South African giraffe) | Moremi Game Reserve, Botswana | MGR9_12 | HG975145  HG975247 | Current paper |
| *G. c. giraffa* (South African giraffe) | Moremi Game Reserve, Botswana | MGR9_13 | HF571170  HF571212 | Fennessy et al. 2013 |
| *G. c. giraffa* (South African giraffe) | Moremi Game Reserve, Botswana | MGR9_14 | HG975146  HG975248 | Current paper |
| *G. c. giraffa* (South African giraffe) | Moremi Game Reserve, Botswana | MGR9_16 | HG975147  HG975249 | Current paper |
| *G. c. giraffa* (South African giraffe) | Moremi Game Reserve, Botswana | MGR9_17 | HG975148  HG975250 | Current paper |
| *G. c. giraffa* (South African giraffe) | Moremi Game Reserve, Botswana | MGR9_18 | HG975149  HG975251 | Current paper |
| *G. c. giraffa* (South African giraffe) | Moremi Game Reserve, Botswana | MGR9_19 | HG975150  HG975252 | Current paper |
| *G. c. giraffa* (South African giraffe) | Moremi Game Reserve, Botswana | MGR9_20 | HG975151  HG975253 | Current paper |
| *G. c. angolensis* (Angolan giraffe) | Unknown | NC012100 | NC012100 | Yasue et al. 2009 unpublished |
| *G. c. giraffa* (South African giraffe) | Nxai Pans, Botswana | NXP9_01 | HF571171  HF571213 | Fennessy et al. 2013 |
| *Okapia johnstoni* | Basel Zoo | Okapi1_0 | HF571214.1  HF571175.1 | Fennessy et al. 2013 |
| *G. c. giraffa* (South African giraffe) | Khamab Kalahari Reserve, South Africa | KKR01 | HG975152  HG975254 | Current paper |
| *G. c. giraffa* (South African giraffe) | Khamab Kalahari Reserve, South Africa | KKR02 | HG975153  HG975255 | Current paper |
| *G. c. giraffa* (South African giraffe) | Khamab Kalahari Reserve, South Africa | KKR03 | HG975154  HG975256 | Current paper |
| *G. c. giraffa* (South African giraffe) | Khamab Kalahari Reserve, South Africa | KKR04 | HG975155  HG975257 | Current paper |
| *G. c. giraffa* (South African giraffe) | Khamab Kalahari Reserve, South Africa | KKR06 | HG975156  HG975258 | Current paper |
| *G. c. giraffa* (South African giraffe) | Khamab Kalahari Reserve, South Africa | KKR07 | HG975157  HG975259 | Current paper |
| *G. c. tippelskirchi* (Masai giraffe) | Selous Game Reserve, Tanzania | SGR01 | HG975158  HG975260 | Current paper |
| *G. c. tippelskirchi* (Masai giraffe) | Selous Game Reserve, Tanzania | SGR05 | HG975159  HG975261 | Current paper |
| *G. c. tippelskirchi* (Masai giraffe) | Selous Game Reserve, Tanzania | SGR11 | HG975160  HG975262 | Current paper |
| *G. c. tippelskirchi* (Masai giraffe) | Selous Game Reserve, Tanzania | SGR12 | HG975161  HG975263 | Current paper |
| *G. c. tippelskirchi* (Masai giraffe) | Selous Game Reserve, Tanzania | SGR13 | HG975162  HG975264 | Current paper |
| *G. c. tippelskirchi* (Masai giraffe) | Selous Game Reserve, Tanzania | SGR14 | HG975163  HG975265 | Current paper |
| *G. c. giraffa* (South African giraffe) | Vumbura Concession, Botswana | V22 | HG975164  HG975266 | Current paper |
| *G. c. giraffa* (South African giraffe) | Vumbura Concession, Botswana | V23 | HG975165  HG975267 | Current paper |
| *G. c. giraffa* (South African giraffe) | Vumbura Concession, Botswana | V24 | HG975166  HG975268 | Current paper |
| *G. c. giraffa* (South African giraffe) | Vumbura Concession, Botswana | V25 | HG975167  HG975269 | Current paper |
| *G. c. giraffa* (South African giraffe) | Vumbura Concession, Botswana | V26 | HG975168  HG975270 | Current paper |
| *G. c. giraffa* (South African giraffe) | Vumbura Concession, Botswana | V27 | HG975169  HG975271 | Current paper |
| *G. c. giraffa* (South African giraffe) | Vumbura Concession, Botswana | V28 | HG975170  HG975272 | Current paper |
| *G. c. giraffa* (South African giraffe) | Vumbura Concession, Botswana | V30 | HG975171  HG975273 | Current paper |
| *G. c. giraffa* (South African giraffe) | Vumbura Concession, Botswana | V31 | HG975172  HG975274 | Current paper |
| *G. c. giraffa* (South African giraffe) | Vumbura Concession, Botswana | V32 | HG975173  HG975275 | Current paper |
| *G. c. giraffa* (South African giraffe) | Vumbura Concession, Botswana | V33 | HG975174  HG975276 | Current paper |
| *G. c. peralta* (West African giraffe) | Niger | WA117 | HG975175  HG975277 | Current paper |
| *G. c. peralta* (West African giraffe) | Niger | WA26 | HG975176  HG975278 | Current paper |
| *G. c. peralta* (West African giraffe) | Niger | WA36 | HG975177  HG975279 | Current paper |
| *G. c. peralta* (West African giraffe) | Niger | WA606 | HG975178  HG975280 | Current paper |
| *G. c. antiquorum* (Kordofan giraffe) | Zakoukma National Park, Chad | ZNP01 | HG975179  HG975281 | Current paper |
| *Antilocapra americana* (Pronghorn) | NN | JN632597 | JN632597 | Hassanin et al. 2012 |
| *Cervus elaphus* (Red deer) | New Zealand | NC007704 | NC007704 | Wada et al. 2006 unpublished |
| *Muntiacus muntjak* (Muntjac) | NN | NC004563 | NC004563 | Shi et al. 2003 unpublished |
| *Pudu puda* (Southern Pudu) | NN | JN632692 | JN632692 | Hassanin et al. 2012 |
| *Rangifer tarandus* (Reindeer) | Hokkaido, Japan | NC007703 | NC007703 | Wada et al. 2006 unpublished |
| *G. c. peralta* (West African giraffe) | Niger | WA609 | HG975180  HG975282 | Current paper |
| *G. c. peralta* (West African giraffe) | Niger | WA612 | HG975181  HG975283 | Current paper |
| *G. c. peralta* (West African giraffe) | Niger | WA614 | HG975182  HG975284 | Current paper |
| *G. c. peralta* (West African giraffe) | Niger | WA619 | HG975183  HG975285 | Current paper |
| *G. c. peralta* (West African giraffe) | Niger | WA621 | HG975184  HG975286 | Current paper |
| *G. c. peralta* (West African giraffe) | Niger | WA622 | HG975185  HG975287 | Current paper |
| *G. c. peralta* (West African giraffe) | Niger | WA623 | HG975186  HG975288 | Current paper |
| *G. c. peralta* (West African giraffe) | Niger | WA628 | HG975187  HG975289 | Current paper |
| *G. c. peralta* (West African giraffe) | Niger | WA631 | HG975188  HG975290 | Current paper |

**References for Supplementary table 1**

Brown DM, Brenneman R, Koepfli KP, Pollinger J, Mila B, Louis EE Jr, Georgiadis N, Grether G, Wayne RK: **Extensive population genetic structure in the giraffe.** *BMC Biol* 2007, **5**:57-70.

Fennessy J, Bock F, Tutchings A, Brenneman R, Janke A: **Mitochondrial DNA analyses show that Zambia's South Luangwa Valley giraffe (*Giraffa camelopardalis thornicrofti*) are genetically isolated.** *Afr J Ecol* 2013, **51**(4):635-640.

Hassanin A, Ropiquet A, Gourmand AL, Chardonnet B, Rigoulet J: **Mitochondrial DNA variability in *Giraffa camelopardalis*: consequences for taxonomy, phylogeography and conservation of giraffes in West and central Africa.** *C R Biol* 2007, **330**:265-274.

Hassanin A, Delsuc F, Ropiquet A, Hammer C, Jansen van Vuuren B, Matthee C, Ruiz-Garcia M, Catzeflis F, Areskoug V, Nguyen TT, Couloux A: **Pattern and timing of diversification of Cetartiodactyla (Mammalia, Laurasiatheria), as revealed by a comprehensive analysis of mitochondrial genomes.** *C R Biol* 2012, **335**(1):32-50.

Shi Y, Li J, Shan X: Muntiacus muntjak mitochondrion, complete genome. 2003. [http://www.ncbi.nlm.nih.gov/nuccore/NC_004563]

Wada K, Nakamura M, Nishibori M, Yokohama M: The complete nucleotide sequence of mitochondrial genome in the reindeer (*Rangifer tarandus*) and red deer (*Cervus elaphus*). 2006, [http://www.ncbi.nlm.nih.gov/nuccore/NC_007704 or http://www.ncbi.nlm.nih.gov/nuccore/NC_007703]

Yasue H, Yamamoto Y, Hayashi T, Honma D, Nishibori N, Nishibori M, Wada Y: *Giraffa camelopardalis* mitochondrial DNA, complete genome. 2001. [http://www.ncbi.nlm.nih.gov/nuccore/AP003424]

Yasue H, Yamamoto Y, Hayashi T, Honma D, Nishibori N, Nishibori M, WadaY: *Giraffa camelopardalis angolensis* mitochondrion, complete genome. 2009. [http://www.ncbi.nlm.nih.gov/nuccore/NC_012100]
